# Asthma-related inflammation promotes lung metastasis of breast cancer cells through CCL11–CCR3 pathway

**DOI:** 10.1186/s12931-021-01652-9

**Published:** 2021-02-19

**Authors:** S. Bekaert, N. Rocks, C. Vanwinge, A. Noel, D. Cataldo

**Affiliations:** 1 Laboratory of Tumor and Development Biology, GIGA-Cancer - University of Liege and CHU Liege, 4000 Liege, Belgium; 2 University of Liege, Tower of Pathology (B23), 3rd Floor, 4000 Liege, Belgium

**Keywords:** Lung metastasis, Asthma, Lung inflammation, CCL11–CCR3, Breast cancer

## Abstract

**Background:**

Mechanisms that preclude lung metastasis are still barely understood. The possible consequences of allergic airways inflammation on cancer dissemination were studied in a mouse model of breast cancer.

**Methods:**

Balb/c mice were immunized and daily exposed to ovalbumin (OVA) from day 21. They were subcutaneously injected with 4T1 mammary tumor cells on day 45 and sacrificed on day 67. Lung metastases were measured by biophotonic imaging (IVIS® 200 Imaging System) and histological measurement of tumor area (Cytomine software). Effects of CCL11 were assessed in vivo by intratracheal instillations of recCCL11 and in vitro using Boyden chambers. CCR3 expression on cell surface was assessed by flow cytometry.

**Results:**

The extent of tumor metastases was significantly higher in lungs of OVA-exposed mice and increased levels of CCL11 expression were measured after OVA exposure. Migration of 4T1 cells and neutrophils was stimulated in vitro and in vivo by recCCL11. 4T1 cells and neutrophils express CCR3 as shown by flow cytometry and a selective CCR3 antagonist (SB-297006) inhibited the induction of 4T1 cells migration and proliferation in response to recCCL11*.*

**Conclusions:**

Allergic inflammation generated by exposure to allergens triggers the implantation of metastatic cells from primary breast tumor into lung tissues plausibly in a CCL11–CCR3-dependent manner. This indicates that asthma related inflammation in lungs might be a risk factor for lung metastasis in breast cancer patients.

## Background

Asthma is one of the most frequent chronic diseases, affecting ∼300 million people worldwide according to The World Health Organization [1]. It is a complex disorder of the respiratory system that causes bronchial inflammation, airway remodeling and hyperresponsiveness [2]. It has been hypothesized that this chronic condition might be a risk factor in developing lung cancer [3], especially in non-smokers [4], and that it might also lead to metastatic dissemination. Indeed, it is now well accepted that microenvironment is an important component in the metastatic process [5]. Based on a Surgical Breast Cancer Database of the Mayo Clinic, in a group of 179 breast cancer patients with recurrence of lung metastases, 29 patients were reported to have had a diagnosis of asthma at least one year prior to the diagnosis of distant metastases, suggesting that this condition may have contributed as a risk factor for lung metastases [6]. Indeed, there are key features associated with asthma-related inflammation and especially the presence of eosinophils. These cells can clearly contribute to anti-cancer immunity by modifying the microenvironment and promoting tumor rejection after T cells infiltration [7], as well as represent indicator of success of therapies against cancer [8]. However, (to date scarce) evidence exists regarding the potential role of eosinophilic inflammation in the establishment of lung metastasis of cancers. Immune cells produce indeed a subset of chemokines [9], especially the cytokine CCL11 which is abundantly produced in the lungs of patients with asthma and also widely expressed in other human tissues including heart, colon, kidney, small intestine, pancreas, liver, and ovaries. CCL11 binds to CCR3, a seven-transmembrane domain that activates a number of signaling pathways. This cytokine is best known as an eosinophil-selective chemoattractant cytokine [10], that might also display effects on neutrophils, [11] although it was recently reported that chemokine receptors (iCCRs) exert no significant effects on neutrophils [12]. Until now, CCL11 implication in cancer progression has been scarcely investigated. CCL11 has also been proposed as a biomarker in different types of cancers with however still little evidence that CCL11 or CCR3 expression may serve as a prognostic factor in cancer [13–15]. Earlier studies showed the role of CCL11 in tumor cells migration, tissue invasion and angiogenesis in ovarian cancer [15]. Chemical inhibitors of CCR3 (SB-297006 or (S)-ethyl-2-benzoylamino-3-(4-nitrophenyl) propionate) have an impact on CCL11-induced migration of mouse neural progenitor cells, as well as on photoreceptor-derived cell line [16–18]. Different cytokines are now therapeutic targets in asthma with different treatments on the market targeting IL5 or IL4 and IL13 receptor and other products in development that comprise CXCR2 antagonists or low molecular weight chemicals that antagonize CCL11 receptor, cysteine-cysteine chemokine receptor-3 (CCR3) [19–21]. As these potent medications allow the control of severe forms of asthma, it is important to unveil any potential link between asthma-related inflammation and possible mechanisms triggering pulmonary metastases.

## Methods

### Cell culture

Murine mammary tumor cells 4T1 (clone 1A4) stably transfected with luciferase gene were purchased from Caliper Life Sciences. Mammary tumor cells were grown at 37 °C, in 5% CO_2_, in Dulbecco’s Modified Eagle Medium (Invitrogen Corp. /Life Technologies, Gent, Belgium) supplemented with 10% fetal bovine serum and 2 mmol l^−1^ L-glutamine, penicillin–streptomycin (100 IU ml^−1^—100 µg ml^−1^) (Invitrogen Corp. /Life Technologies).

### Mice

Male Balb/cJRj mice, 6 to 8 weeks old, were purchased from Charles River (Cologne, Germany). All animal experimental procedures were approved by the ethical committee of the University of Liège. Food and water were supplied ad libitum.

### Experimental asthma protocol

In the OVA-induced inflammation model, mice were immunized by i.p. injection of OVA (10 μg; Sigma-Aldrich, Schnelldorf, Germany) and aluminum hydroxide on days 1 and 11. From days 21 to 67, mice were exposed to inhalation of 1% OVA or PBS for 30 min. Mice challenged with PBS were used as controls (control littermates). At day 45, luciferase-expressing 4T1 cells (2 × 10^5^ cells/200 μl of serum-free medium) were subcutaneously injected into flanks of mice (n = 8 mice/group). Volume of primary tumor growth was evaluated on days 20, 23 and 25 with a caliper. In vivo*,* lung metastases were quantified by bioluminescence measurement after intraperitoneal injection (i.p) of D-luciferin (150 mg/kg in PBS; Promega, Madison, WI) using the IVIS® 200 Imaging System (Caliper Life Sciences, Hopkinton, MA). Quantitative assessment of lung metastasis was performed by determining “regions of interest” (ROI) by measuring bioluminescence intensity using Living Image software.

### Experimental CCL11 protocol

Mice (n = 6/group) were anesthetized with 2.5% isoflurane/oxygen mixture and intratracheally (i.t) injected with Phosphate Buffered Saline (PBS) or recombinant CCL11 (10 ng/ml PBS, R&D System, MN), 3 times per week. After 4 i.t instillations, murine mammary cancer 4T1 cells (1 × 10^5^ cells/50 μl of serum-free medium) were injected into the tail vein of Balb/c mice.

At the end of each experimental protocol, animals were sacrificed and bronchoalveolar lavage fluid (BALF) was performed via intratracheal instillation of 4 × 1 ml PBS-EDTA 0.05 mM solution (Calbiochem, Darmstadt, Germany). BALF supernatant was collected for protein assessment while cells were used for differential cell counts. Differential cell counts based on morphologic criteria were carried out on cytocentrifuged preparations after staining with haematoxylin–eosin (Diff-Quick, Dade, Belgium).

### Pulmonary histology

Left main bronchus was clamped, excised and preserved at -80ºC. The right lung was infused at a pressure of 25 cm with 4% paraformaldehyde and embedded in paraffin. Six sections of 5 μm were randomly sectioned and stained with hematoxylin and eosin (H.E). Each subsequent section was spaced 50 μm from the previous one. Lung tumor development was evaluated by measuring lung tumor area and reporting it to the total area of lung tissues analyzed, on eight randomly selected H.E sections per animal in each experimental group using the open source Cytomine software (http://www.cytomine.be, GIGA, Liège University Research Center, Belgium). This software implements a machine learning algorithm which provides automatic tumor detections that were manually reviewed and edited by an experimented observer blinded to experimental details [22].

### Measurement of cytokines by Elisa

CCL11 ELISA kits were used according to the manufacturer’s instructions (Duoset; R&D Systems McKinley Pl NE, MN).

### Chemotaxis assay

Chemotaxis assay was performed using 24-Well transwell plates (Corning® Costar®). In the lower well of the Boyden chamber assay, Bovine serum albumin (BSA) 1% diluted in DMEM (Life Technologies, Gent, Belgium) supplemented with 1% FBS was used as control. Recombinant CCL11 (100 ng/ml) diluted in DMEM BSA 1% and FBS 1% supplemented or not with CCR3 antagonist (SB-297006; 1 µM) was used to measure chemotactic potential. In the upper well, 4T1 cells (1 × 10^5^ cells/well) or neutrophils (4 × 10^5^ cells/well) were loaded, in serum-free DMEM supplemented with BSA 0.1%, on polycarbonate filter pore size of 8 µm or 5 µm respectively. After a period of incubation of 16 h at 37 °C for tumor cells and 4 h for neutrophils, cells were fixed (Methanol, -20 °C) and stained with Giemsa 4%. The migrated cells on the reverse side of the filter were mounted on slide, digitized (Hamamatsu, NanoZoomer 2.0-HT series, Shizuoka, Japan) and quantified with Image J software (National Institutes of Health, Bethesda, MA). Migrating cells were counted on eight random fields and the average value was used as an individual score for each membrane.

### Mouse neutrophils isolation

In order to recover lung neutrophils using the "FACS aria", mice (n = 6) received intra-tracheal instillations of recombinant CCL11 (10 ng) on J0, J2, J5, and J7 and were sacrificed on J8. Lungs were incubated for 45 min with shaking at 37 °C in medium without serum and IV collagenase (1 mg/ml) (Gibco, Belgium). After homogenization, FBS was added in equal volume to neutralize collagenase IV. Samples were centrifuged at 335 g for 5 min and treated by addition of "Red Blood Cell Lysing Buffer" (Sigma, Germany). The samples were then separated into several phases on Histopaque® medium (Sigma, Germany). The granulocyte phase was stored and washed in PBS 2% FBS. Neutrophils ("high" SSC; "low" FSC) were labeled with antibodies (CD45^+^, CD11b^+^, Gr1^+^, CCR3^+^). On the basis of these markers, cells were isolated using the "FACS Aria with a purity of 93.92%. Once recovered, the cells were fixed on a slide (CytoSpin; Statspin Cytofuge 2; Iris, USA) to confirm they are neutrophils.

### Flow cytometry

Mice lungs were dissected and incubated in a digestion medium containing RPMI 1640 (Roswell Park Memorial Institute; Lonza, Braine-l’Alleud, Belgium) supplemented with 1 mg/ml collagenase type IV (Life Technologies, Gent, Belgium) and 20 µg/ml DNase I (grade II from bovine pancreas; Roche, Vilvoorde, Belgium). Recovered cells were incubated with FcR blocking antibody (anti-CD16/CD32, clone 2.4G2, B&D Biosciences, Erembodegem, Belgium) to reduce nonspecific binding. 4T1 cells were also labelled with Annexin FITC-PI to determine cell viability. The following antibodies were used to identify mouse granulocytes subpopulations: V450 Horizon-conjugated anti-CD45, FITC-conjugated anti-CCR3, PE-conjugated anti-CD11b, PerCP-CY5.5-conjugated anti-GR1 (B&D Biosciences, Erembodegem, Belgium). Flow cytometry data acquisition was performed on FACS Canto II (B&D Biosciences) where 1 × 10^6^ events were analyzed per sample and BD FACS Diva software was used for data analysis.

### Proliferation assay

The Cell Proliferation ELISA BrdU (colorimetric) assay Kit (Roche Applied Science, Vilvoorde, Belgium.) was used to evaluate cell proliferation of 4T1 cells according to the manufacturer’s protocol. 4T1 cells (2000 cells/well) were plated on a 96-well plate in DMEM 1% FBS (control), CCL11 recombinant (100 ng/ml) supplemented or not with CCR3 antagonist (SB-297006; 1 µM) for 72 h.

### Statistical analysis

Reported values are expressed as mean ± SEM. Statistical analysis differences between experimental groups were assessed using Instat software (GraphPad). Data were analyzed by Student’s t-test or one-way Anova. Variations were considered to be statistically significant at a *p value < 0.05.

## Results

### Allergen exposure promotes migration of breast cancer cells to the lungs

**Fig. 1:**
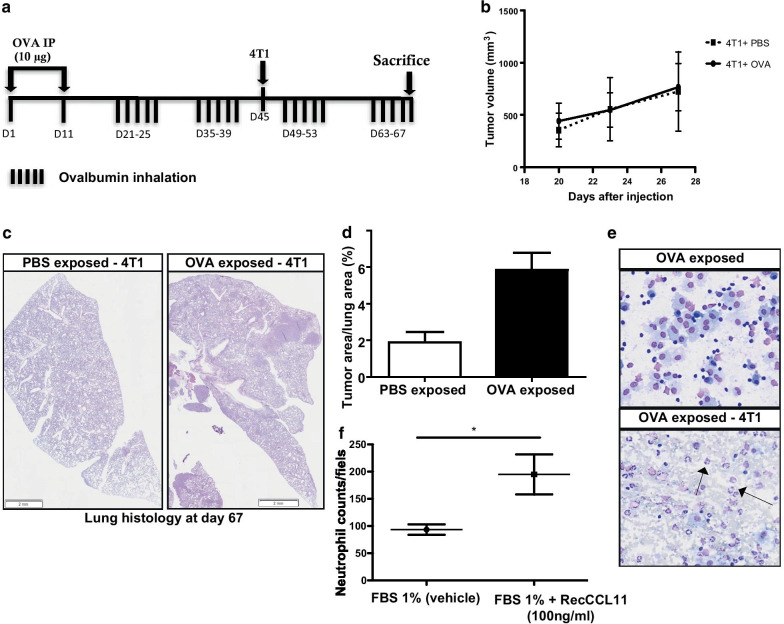
Inflammation generated by OVA challenge induces tumor cell dissemination to the lung parenchyma (n = 8/group). **a** Timeline of mice challenged with OVA and s.c injected with 4T1 cells. **b** Volume of subcutaneous primary tumor. **c**, **d** Tumor size quantification assessed by measuring the ratio between the area of tumor foci in lungs and total lung tissue area on 8 sections per mouse in each group. Scale bar: 2 mm (hematoxylin–eosin). Results are expressed as mean tumor area/lung area ± SEM and are representative of 2 individual experiments. OVA indicate ovalbumine; PBS, phosphate-buffered saline (**e**) representative BALF total cell of mice exposed to OVA and subcutaneously injected with 4T1 cells. Scale bar: 50 µm (hematoxylin–eosin). **f** Quantification of isolated neutrophils migrating to a gradient of recCCL11 during 4 h using Boyden chamber assay. Neutrophils migration was estimated on 16 random fields (20 ×) of triplicate wells.*p < 0.05

In order to analyze the effects of inflammation elicited by allergen exposure on metastatic dissemination of tumor cells, Balb/c mice were sensitized and exposed to inhaled OVA or PBS from day 21 to 67 and were subcutaneously (s.c) injected with 4T1 cancer cells (murine breast cancer cells of Balb/c background) on day 45 (Fig. 1a). While no differences were observed between groups regarding subcutaneous primary tumor growth (Fig. 1b), histology performed at the time of sacrifice confirmed that area occupied by metastases in lungs of mice exposed to OVA aerosols was higher as compared to PBS-exposed animals (n = 8, Fig. 1c, d).

### CCL11 levels are elevated in BALF after allergen exposure and contribute to lung inflammation

Analysis of BALF of mice challenged with OVA showed predominantly macrophages and eosinophils. In mice challenged with OVA and s.c injected with 4T1 cells (n = 8), elevated amounts of eosinophils and neutrophils were measured (Fig. 1e; Table 1). CCL11 is a key mediator in asthma and levels of this protein were measured by Elisa in lung tissue extracts. OVA-challenged animals displayed significantly higher CCL11 levels as compared to control mice (Table 1). 4T1 cells extracts contained detectable levels of CCL11 as measured by Elisa (18.68 pg/ml of cell extract). In an *ex-vivo* experiment in Boyden chambers, recCCL11 significantly increased neutrophils migration (after 4 h) as compared to PBS (Fig. 1f *p < 0.05). To confirm the influence of recCCL11 on lung neutrophils migration in vivo, naïve BALB/c mice were IT instilled with recCCL11. After 24 h, CCL11-treated animals displayed large amounts of neutrophils in BALF (19.8% ± 4.43; n = 3).

**Table 1:** Differential cell counts (percentages) in the bronchoalveolar lavage fluid after treatment with PBS or OVA inhalation and subcutaneous injection of 4T1 cells

Differential cell counts				
n = 8	INH		INH + 4T1 subcutaneous injection	
Cell type	PBS	OVA	PBS	OVA
Eosinophils	3.53 ± 0.39	29.5 ± 5.74	11 ± 1.86	17.3 ± 5.15
Neutrophils	6.1 ± 1.43	5.46 ± 1.39	27.35 ± 1.74	46.31 ± 5.16**
Lymphocytes	0.10 ± 0.10	0.0 ± 0.0	0.0 ± 0.0	0.77 ± 0.43
Epithelial cells	5.83 ± 1.93	5.13 ± 1.72	1.62 ± 0.69	2.81 ± 0.97
Macrophages	84.3 ± 2.8	59.7 ± 4.3*	59.9 ± 2.76	32.63 ± 6.6**

Proteins level expression of CCL11				
n = 8	INH		INH + 4T1 subcutaneous injection	
CCL11 (pg/ml)	PBS	OVA	PBS	OVA
Mean ± SEM	0,21 ± 0,04	0,58 ± 0,12*	0,36 ± 0,07	0,42 ± 0,08

Proteins level expression of CCL11 (pg/ml) in lung of mice exposed to PBS or OVA and subcutaneously injected with 4T1 cells. *p < 0.05, **p < 0.01.

### CCL11 promotes tumor cells migration to lung parenchyma

**Fig. 2:**
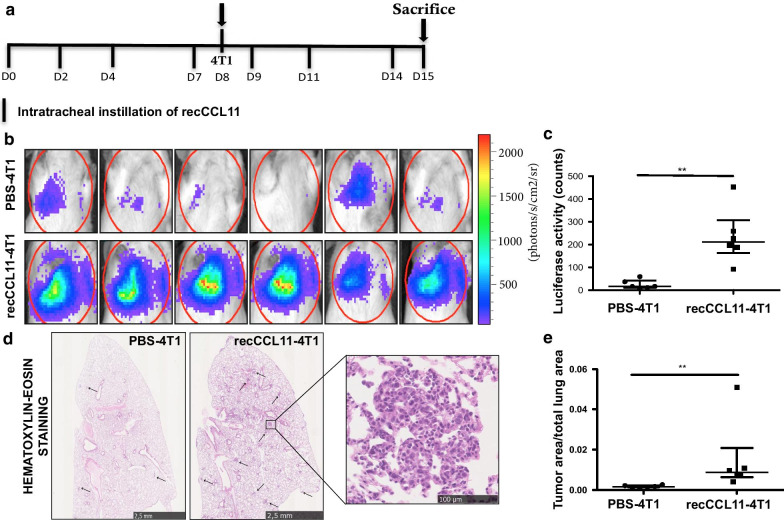
Intratracheal injection of CCL11 recombinant (recCCL11) induces 4T1 tumor cell migration to lung tissue (n = 6/group). **a** Timeline of experiment. Mice were challenged with i.t of recCCL11 and i.v injected with luciferase-stably transfected 4T1 cells. **b**, **c** On day 15, biophotonic monitoring of lung metastasis in animals and quantification of bioluminescence in regions of interest (ROI) determined around lungs. **d**, **e** Representative hematoxylin–eosin–stained sections of lung tissues and tumor size quantification assessed by measuring the ratio between the area of tumor foci in lungs and total lung tissue area on 8 sections per mouse in each group. Scale bar: 2.5 mm, 100 µm. Results are expressed as mean tumor area/lung area ± SEM. **p < 0.01

To evaluate whether CCL11 might promote tumor cells migration to lung parenchyma, mice were intravenously injected with 4T1 cells after intratracheal instillation of recombinant CCL11 (recCCL11) (Fig. 2a). Luciferase activity in lungs corresponding to the presence of 4T1 cells and measured the day of sacrifice (J + 6) was significantly higher in recCCL11-treated mice as compared to the corresponding control groups (Fig. 2b, c; **p < 0.01). Histological analysis confirmed that the area occupied by metastases in the lungs of recCCL11-treated mice was significantly higher as compared to PBS-instilled mice (Fig. 2c, d; n = 6; **p < 0.01). Elevated neutrophil counts were measured in BALF of recCCL11-treated mice subcutaneously injected with tumor cells (Table 2).

**Table 2:** Differential cell counts (percentages) in the bronchoalveolar lavage fluid after treatment with PBS or recCCL11 and intravenous injection of 4T1 cells

n = 6	IT + intravenous injection	
Cell type	PBS	CCL11
Eosinophils	0.65 ± 0.33	5.25 ± 1.06*
Neutrophils	5.96 ± 1.5	52.98 ± 1.72***
Lymphocytes	0.0 ± 0.0	0.0 ± 0.0
Epithelial cells	12.08 ± 3.45	2.36 ± 0.99*
Macrophages	81.18 ± 3.2	39.28 ± 2.13***

*p < 0.05, ***p < 0.001

### CCL11–CCR3 interaction induces 4T1 cells migration and proliferation

**Fig. 3:**
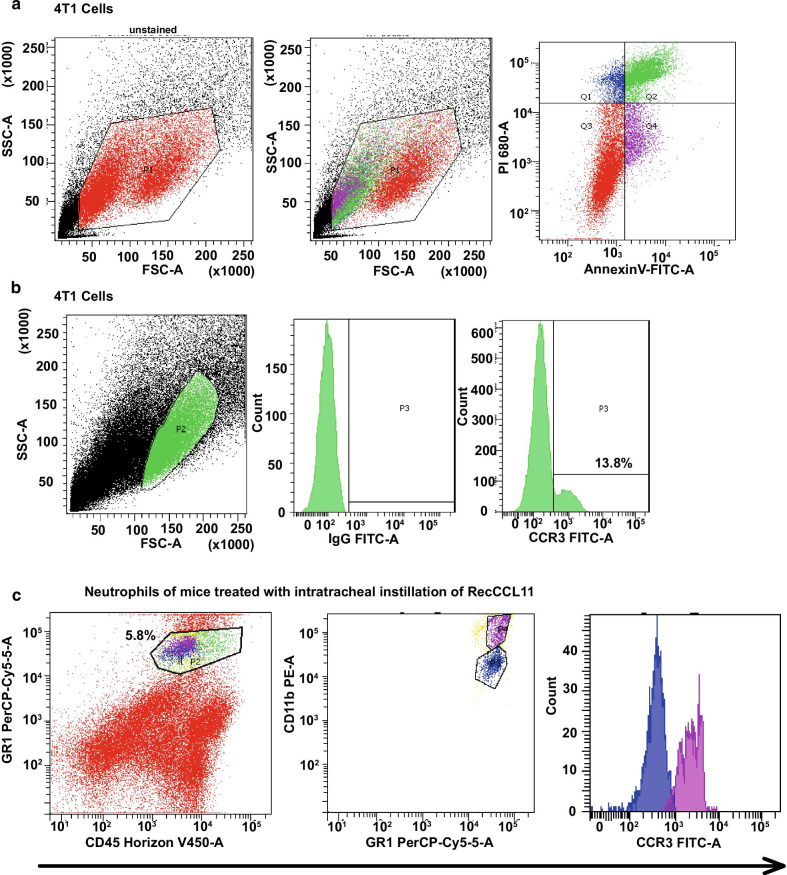
Measurement of CCR3 on 4T1 cells and neutrophils by flow cytometry. **a** The dot plot diagrams represent typical apoptotic and necrotic 4T1 cell populations detected by Annexin V-FITC and PI staining. The lower left quadrants (Q3; 49.2%) of the panels show viable intact cells, which were negative for Annexin V-FITC binding and excluded PI staining (FITC-/PI-); the upper right quadrants (Q1; 7.5%, Q2; 32.1%) show nonviable, necrotic cells, which were positive for Annexin V-FITC binding and PI uptake (FITC + /PI +). The lower right quadrants (Q4; 11.2%) represent apoptotic cells, positive for Annexin V-FITC and negative for PI (FITC + /PI-). **b** Representative histograms for flow cytometric analysis of surface expression of CCR3 receptors on 4T1 cells. IgG: Isotype control. **c** Lung neutrophils of mice treated with intratracheal instillation of recCCL11, sorted by FACS Aria

**Fig. 4:**
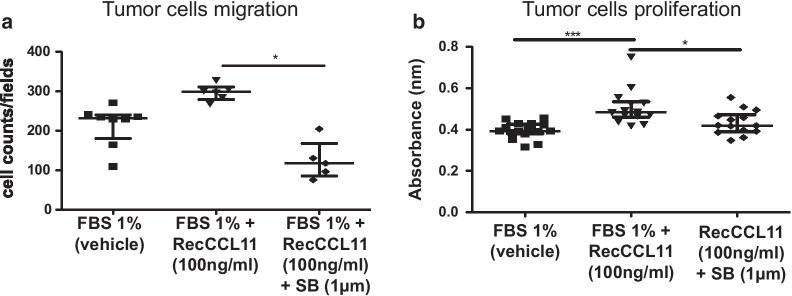
Measurement of CCR3 antagonist (SB-297006) effects on 4T1 cells migration and proliferation. **a** Quantification of 4T1 tumor cells chemotaxis, using Boyden chamber assay, supplemented with recCCL11 and CCR3 antagonist (FBS1%: n = 8. FBS 1% + RecCCL11: n = 6 and FBS 1% + RecCCL11 + SB: n = 5), after 16 h. **b** After 72 h, proliferation of 4T1 cells treated with recCCL11 and CCR3 antagonist was assessed using BrdU staining (FBS 1%: n = 16, FBS 1% + RecCCL11: n = 14 and FBS 1% + RecCCL11 + SB: n = 14). Results are expressed as mean ± SEM. *p < 0.05, ***p < 0.001

Both 4T1 tumor cells and neutrophils isolated from lung parenchyma displayed significant CCR3 expression as measured by FACS (Fig. 3a-c). The strategy used Annexin V signal that provides a very sensitive method for detecting cellular apoptosis, while propidium iodide (PI) was used to detect necrotic or late apoptotic cells, characterized by the loss of integrity of plasma and nuclear membranes. This cellular staining allowed discriminating viable cells which are negative with both probes (PI/FITC -/-; Q3). On this basis, it is possible to differentiate the percentage of viable 4T1 tumor cells (13.8%) expressing the CCR3 receptor on their surface. Furthermore, in order to obtains counts of pulmonary neutrophils, lung lysate of mice treated with intratracheal instillation of recCCL11 was sorted by FACS Aria according to the CD45 + / GR1 + / CD11b^hi^ labeling. In this population, neutrophils positive for CCR3 have been discriminated (31.5%). Inhibition experiments were performed in vitro in order to understand whether 4T1 cells migration and proliferation were driven by a CCL11–CCR3 interaction. Interestingly, tumor cells migration measured in Boyden chambers was decreased when culture medium was supplemented with the CCR3 antagonist SB-297006 (Fig. 4a; *p < 0.05). Proliferation of tumor cells was significantly reduced after 72 h arguing for a direct effect of CCL11 on CCR3 that in turn promotes cell proliferation (Fig. 4b; *p < 0.05).

## Discussion

This study with potential important clinical implications was performed in order to identify potential mechanisms that might explain how pre-existing lung parenchyma allergen-driven inflammation could enhance metastasis. We previously reported that elevated lung levels of PGP tripeptide (collagen fragments produced after collagen cleavage by inflammatory cells-derived MMP9) trigger lung metastasis [23]. Other authors also reported about the potential role of pro-inflammatory mediators in tumor progression and genesis of metastases [24]. In this study, the main purpose was to verify the hypothesis that allergen-induced inflammation has the capacity to generate a burden of cancer cell metastasis in lung parenchyma. Indeed, to the best of our knowledge, there is no study to date reporting a correlation between asthma and lung metastasis of breast cancer, while some epidemiological studies suggest that allergic diseases might be associated with an increased risk of breast cancer [25]. Moreover, epidemiological studies suggest an increased risk of lung cancer as a possible consequence of changes in lung microenvironment promoted by asthma-related inflammation [26–28]. In the present study, we report that OVA-generated inflammation triggers the implantation of metastatic cells into lung tissue, probably in a CCL11–CCR3–dependent manner. This is indeed supported by (i) the measurement of increased cancer cells dissemination to lungs of Balb/c mice immunized and challenged with inhaled ovalbumin; (ii) the higher expression of CCL11 in lungs of mice OVA-challenged and subcutaneously injected with 4T1 cells; (iii) the stimulation of neutrophils and 4T1 cells migration by the use of rec CCL11 in vitro and in vivo; (iv) the flow cytometry studies showing that CCR3 is expressed by 4T1 cells and neutrophils; (v) the abolishment of CCL11-induced 4T1 cells migration and proliferation when cells are incubated with a CCR3 selective antagonist. Our results are concordant with a study showing that allergen-induced pulmonary inflammation resulted in an increase in lung metastases of B16F10 melanoma cells [6] and we propose a mechanism based on the activation of the CCL11–CCR3 axis.

Asthma is a chronic inflammatory disorder resulting from a complex interaction between inflammatory cells, mediators and airway structural cells. CCL11 is one of the key cytokines of the cascade of inflammatory mediators leading to an established allergen-driven airway inflammation. Importantly, we demonstrate for the first time that 4T1 cells express CCR3 and are in vitro and in vivo sensitive to CCL11.

In our experimental setting, we cannot rule out that other mechanisms or other cell types could play a role in the increased dissemination of cancer cells. Hence, mast cells activation during anaphylaxis was reported to increase mouse melanoma cells dissemination in an HDAC3-dependent manner [29] and mast cells have been shown to increase growth of Hodgkin lymphoma tumors and plasma cell tumors [30, 31]. Also, NETs-producing neutrophils also play crucial roles in cancer dissemination to the lung [32]. Recent studies performed on mice depleted for iCCRs did show that these receptors are not involved in neutrophils recruitment in acute inflammatory conditions [12]. The increased number of neutrophils in the BAL of mice injected with 4T1 is expected and not modified whether mice were exposed to OVA or PBS. Indeed, 4T1 mammary tumors were reported to induce an accumulation of neutrophils in various organs including lung parenchyma [33]. This has been reported previously by different authors and it was shown that these cells produce the c-Kit ligand, SCF and are able to expand a specific population of myeloid cells (Ly6Ghi Ly6Clow c-Kit +) [34, 35]. The relationship between neutrophils and cancer dissemination is complex. Indeed, our group previously reported that 4T1 cells produce a soluble factor inducing the production of IL16 by neutrophils and, in turn, this cytokine promotes in vitro 4T1 cell adhesiveness, invasiveness, and migration [33].

In conclusion, we report that allergen-induced inflammation triggers metastatic dissemination to lung parenchyma by activating the CCL11–CCR3 pathway. This should be assessed in human in observational cohorts and by retrospective analysis of results of clinical trials.

## Data Availability

Data sharing not applicable to this article as no datasets were generated or analyzed during the current study.

## References

[1] Masoli M, Fabian D, Holt S, Beasley R (2004). Global Initiative for Asthma P: the global burden of asthma: executive summary of the GINA Dissemination Committee report. Allergy.

[2] Jacobsen EA, Ochkur SI, Doyle AD, LeSuer WE, Li W, Protheroe CA, Colbert D, Zellner KR, Shen HH, Irvin CG (2017). Lung pathologies in a chronic inflammation mouse model are independent of eosinophil degranulation. Am J Respir Crit Care Med.

[3] Rosenberger A, Bickeboller H, McCormack V, Brenner DR, Duell EJ, Tjonneland A, Friis S, Muscat JE, Yang P, Wichmann HE (2012). Asthma and lung cancer risk: a systematic investigation by the International Lung Cancer Consortium. Carcinogenesis.

[4] Garcia Sanz MT, Gonzalez Barcala FJ, Alvarez Dobano JM, Valdes Cuadrado L (2011). Asthma and risk of lung cancer. Clin Transl Oncol.

[5] Hanahan D, Weinberg RA (2011). Hallmarks of cancer: the next generation. Cell.

[6] Taranova AG, Maldonado D, Vachon CM, Jacobsen EA, Abdala-Valencia H, McGarry MP, Ochkur SI, Protheroe CA, Doyle A, Grant CS (2008). Allergic pulmonary inflammation promotes the recruitment of circulating tumor cells to the lung. Cancer Res.

[7] Carretero R, Sektioglu IM, Garbi N, Salgado OC, Beckhove P, Hammerling GJ (2015). Eosinophils orchestrate cancer rejection by normalizing tumor vessels and enhancing infiltration of CD8(+) T cells. Nat Immunol.

[8] Ferrucci PF, Gandini S, Cocorocchio E, Pala L, Baldini F, Mosconi M, Antonini Cappellini GC, Albertazzi E, Martinoli C (2017). Baseline relative eosinophil count as a predictive biomarker for ipilimumab treatment in advanced melanoma. Oncotarget.

[9] Garcia G, Godot V, Humbert M (2005). New chemokine targets for asthma therapy. Curr Allergy Asthma Rep.

[10] Garcia-Zepeda EA, Rothenberg ME, Ownbey RT, Celestin J, Leder P, Luster AD (1996). Human eotaxin is a specific chemoattractant for eosinophil cells and provides a new mechanism to explain tissue eosinophilia. Nat Med.

[11] Menzies-Gow A, Ying S, Sabroe I, Stubbs VL, Soler D, Williams TJ, Kay AB (2002). Eotaxin (CCL11) and eotaxin-2 (CCL24) induce recruitment of eosinophils, basophils, neutrophils, and macrophages as well as features of early- and late-phase allergic reactions following cutaneous injection in human atopic and nonatopic volunteers. J Immunol.

[12] Dyer DP, Medina-Ruiz L, Bartolini R, Schuette F, Hughes CE, Pallas K, Vidler F, Macleod MKL, Kelly CJ, Lee KM (2019). Chemokine Receptor Redundancy and Specificity Are Context Dependent. Immunity.

[13] Heidegger I, Hofer J, Luger M, Pichler R, Klocker H, Horninger W, Steiner E, Jochberger S, Culig Z (2015). Is Eotaxin-1 a serum and urinary biomarker for prostate cancer detection and recurrence?. Prostate.

[14] Johrer K, Zelle-Rieser C, Perathoner A, Moser P, Hager M, Ramoner R, Gander H, Holtl L, Bartsch G, Greil R, Thurnher M (2005). Up-regulation of functional chemokine receptor CCR3 in human renal cell carcinoma. Clin Cancer Res.

[15] Levina V, Nolen BM, Marrangoni AM, Cheng P, Marks JR, Szczepanski MJ, Szajnik ME, Gorelik E, Lokshin AE (2009). Role of eotaxin-1 signaling in ovarian cancer. Clin Cancer Res.

[16] Wang F, Baba N, Shen Y, Yamashita T, Tsuru E, Tsuda M, Maeda N, Sagara Y (2017). CCL11 promotes migration and proliferation of mouse neural progenitor cells. Stem Cell Res Ther.

[17] Kuse Y, Tsuruma K, Kanno Y, Shimazawa M, Hara H (2017). CCR3 Is Associated with the Death of a Photoreceptor Cell-line Induced by Light Exposure. Front Pharmacol.

[18] White JR, Lee JM, Dede K, Imburgia CS, Jurewicz AJ, Chan G, Fornwald JA, Dhanak D, Christmann LT, Darcy MG (2000). Identification of potent, selective non-peptide CC chemokine receptor-3 antagonist that inhibits eotaxin-, eotaxin-2-, and monocyte chemotactic protein-4-induced eosinophil migration. J Biol Chem.

[19] O'Byrne PM, Naji N, Gauvreau GM (2012). Severe asthma: future treatments. Clin Exp Allergy.

[20] Song DJ, Shim MH, Lee N, Yoo Y, Choung JT (2017). CCR3 Monoclonal Antibody Inhibits Eosinophilic Inflammation and Mucosal Injury in a Mouse Model of Eosinophilic Gastroenteritis. Allergy Asthma Immunol Res.

[21] Tian M, Chen L, Ma L, Wang D, Shao B, Wu J, Wu H, Jin Y (2016). Expression and prognostic significance of CCL11/CCR3 in glioblastoma. Oncotarget.

[22] Marée R, Rollus L, Stevens B, Louppe G, Caubo O, Rocks N, Bekaert S, Cataldo D, Wehenkel L: *A hybrid human-computer approach for large-scale image-based measurements using Web services and machine learning.* 2014.

[23] Bekaert S, Fillet M, Detry B, Pichavant M, Maree R, Noel A, Rocks N, Cataldo D (2017). Inflammation-Generated Extracellular Matrix Fragments Drive Lung Metastasis. Cancer Growth Metastasis.

[24] Wu Y, Zhou BP (2009). Inflammation: a driving force speeds cancer metastasis. Cell Cycle.

[25] Sadeghi F, Shirkhoda M (2019). Allergy-Related Diseases and Risk of Breast Cancer: The Role of Skewed Immune System on This Association. Allergy Rhinol (Providence).

[26] Vesterinen E, Pukkala E, Timonen T, Aromaa A (1993). Cancer incidence among 78,000 asthmatic patients. Int J Epidemiol.

[27] Qu YL, Liu J, Zhang LX, Wu CM, Chu AJ, Wen BL, Ma C, Yan XY, Zhang X, Wang DM (2017). Asthma and the risk of lung cancer: a meta-analysis. Oncotarget.

[28] Kantor ED, Hsu M, Du M, Signorello LB (2019). Allergies and Asthma in Relation to Cancer Risk. Cancer Epidemiol Biomarkers Prev.

[29] Eom S, Kim Y, Park D, Lee H, Lee YS, Choe J, Kim YM, Jeoung D (2014). Histone deacetylase-3 mediates positive feedback relationship between anaphylaxis and tumor metastasis. J Biol Chem.

[30] Mizuno H, Nakayama T, Miyata Y, Saito S, Nishiwaki S, Nakao N, Takeshita K, Naoe T (2012). Mast cells promote the growth of Hodgkin's lymphoma cell tumor by modifying the tumor microenvironment that can be perturbed by bortezomib. Leukemia.

[31] Nakayama T, Yao L, Tosato G (2004). Mast cell-derived angiopoietin-1 plays a critical role in the growth of plasma cell tumors. J Clin Invest.

[32] Rocks N, Vanwinge C, Radermecker C, Blacher S, Gilles C, Maree R, Gillard A, Evrard B, Pequeux C, Marichal T (2019). Ozone-primed neutrophils promote early steps of tumour cell metastasis to lungs by enhancing their NET production. Thorax.

[33] Donati K, Sepult C, Rocks N, Blacher S, Gerard C, Noel A, Cataldo D (2017). Neutrophil-Derived Interleukin 16 in Premetastatic Lungs Promotes Breast Tumor Cell Seeding. Cancer Growth Metastasis.

[34] Pan PY, Wang GX, Yin B, Ozao J, Ku T, Divino CM, Chen SH (2008). Reversion of immune tolerance in advanced malignancy: modulation of myeloid-derived suppressor cell development by blockade of stem-cell factor function. Blood.

[35] Kuonen F, Laurent J, Secondini C, Lorusso G, Stehle JC, Rausch T, Faes-Van't Hull E, Bieler G, Alghisi GC, Schwendener R (2012). Inhibition of the Kit ligand/c-Kit axis attenuates metastasis in a mouse model mimicking local breast cancer relapse after radiotherapy. Clin Cancer Res.

